# Supplementary material for: Pseudolesion in the right parafissural liver parenchyma on CT: The base is found in embryology and collagen content
Source: PLoS One. 2020 Jan 27;15(1):e0221544. doi: 10.1371/journal.pone.0221544 (PMC6984698; doi:10.1371/journal.pone.0221544)
Supplement: S1 File — (DOCX) [file pone.0221544.s002.docx]

**Author list:**

Willemijn M Klein

Roles: **Conceptualization, Data Curation, Formal Analysis, Investigation, Methodology, Project Administration, Supervision, Validation, Writing – Original Draft Preparation, Writing – Review & Editing**

Affiliation: Dept of Radiology and Nuclear Medicine, Radboud university medical center, Nijmegen, the Netherlands

Lianne JP Sonnemans

Roles: **Data Curation, Formal Analysis, Investigation, Methodology, Project Administration, Validation, Writing – Original Draft Preparation, Writing – Review & Editing**

Affiliation: Dept of Radiology and Nuclear Medicine, Radboud university medical center, Nijmegen, the Netherlands

Sabine Franckenberg

Roles: **Data Curation, Investigation, Project Administration, Resources, Writing – Review & Editing**

Affiliation: Institute for Diagnostic and Interventional Radiology, University Hospital Zurich, University of Zurich, Rämistrasse 100, 8091 Zürich, Switzerland (current working address)

Barbara Fliss

Roles: **Data Curation, Investigation, Project Administration, Resources, Writing – Review & Editing**

Affiliation: Department of Forensic Medicine and Imaging, Institute of Forensic Medicine, University of Zürich, Winterthurerstrasse 190/57, 8057 Zürich, Switzerland

Dominic Gascho

Roles: **Data Curation, Investigation, Project Administration, Resources, Writing – Review & Editing**

Affiliation: Department of Forensic Medicine and Imaging, Institute of Forensic Medicine, University of Zürich, Winterthurerstrasse 190/57, 8057 Zürich, Switzerland

Mathias Prokop

Roles: **Conceptualization, Formal Analysis, Funding Acquisition, Methodology, Resources, Supervision, Validation, Writing – Original Draft Preparation, Writing – Review & Editing**

Affiliation: Dept of Radiology and Nuclear Medicine, Radboud university medical center, Nijmegen, the Netherlands

Wouter H Lamers

Roles: **Investigation, Methodology, Supervision, Writing – Original Draft Preparation, Writing – Review & Editing**

Affiliation: Department of Anatomy and Embryology, Maastricht University, Maastricht, the Netherlands

Jill PJM Hikspoors

Roles: **Investigation, Methodology, Validation, Writing – Original Draft Preparation, Writing – Review & Editing**

Affiliation: Department of Anatomy and Embryology, Maastricht University, Maastricht, the Netherlands

Michael J Thali

Roles: **Conceptualization, Funding Acquisition, Resources, Supervision, Writing – Review & Editing**

Affiliation: Department of Forensic Medicine and Imaging, Institute of Forensic Medicine, University of Zürich, Winterthurerstrasse 190/57, 8057 Zürich, Switzerland

Patricia M Flach

Roles: **Conceptualization, Funding Acquisition, Investigation, Methodology, Project Administration, Resources, Supervision, Validation, Writing – Original Draft Preparation, Writing – Review & Editing**

Affiliation: Department of Forensic Medicine and Imaging, Institute of Forensic Medicine, University of Zürich, Winterthurerstrasse 190/57, 8057 Zürich, Switzerland

Clinic for Radiology and Nuclear Medicine, Cantonal Hospital St. Gallen, Haus 03, Rorschacher Strasse 95, 9007 St. Gallen, Switzerland (current working address)

# Supporting information

**Figure 1**. **Biopsy locations.** Postmortem CT slice through the liver. The arrows indicate the site of the biopsies on the left and right site of the falciform ligament.

**Figure 2.** **Case A with a pseudolesion.** Axial slice of the non contrast-enhanced postmortem CT scan, through the liver of a 54 year old woman who died of natural cardiac cause. CT attenuation values and collagen and fat content on the right parafissural side (green arrow) were 39 HU, 1.17% and 0.51% respectively, compared to 64 HU, 0.68% and 0.53% on the left side (white arrow).

**Figure 3. Case B with a pseudolesion.** Axial slice of the non contrast-enhanced postmortem CT scan, through the liver of a deceased 32 year old female (case B) who died of drug intoxication. The right parafissural side (green arrow) shows a pseudolesion with low attenuation compared to the left parafissural side (white arrow) The purple arrow indicates a paraumbilical vein.

**Figure 4. Embryonic liver.** Histological section (fig.4A) and 3D model (fig.4B) of the human embryonic liver aged 34 days. The open purple arrow indicates the involuting right umbilical vein; the solid purple arrow the left umbilical vein. The black dotted line marks the connection of the ventral side of the liver to the ventral body wall, which hardly changes over time and becomes a thin structure (falciform ligament) as the volume of the liver increases about 40-fold between 5 and 8 weeks of development. GB – Gallbladder, ST – Stomach, PS – Portal sinus. Color code: blue – inferior caval vein, light blue – portal vein, grey – Gastro-intestinal tract.

**Figure 5. Adult liver.** Caudal (fig.5.A) and ventral (fig.5.B) view on 3D reconstructions of a contrast-enhanced CT of an adult liver (in transparent brown). The pseudolesion on the right parafissural side is indicated in green (green arrow). The purple arrow indicates a paraumbilical vein of Sappey, which lies in the falciform ligament. GB -gallbladder, in dark green. PS - portal sinus, in light blue. Inferior caval vein, in dark blue.

**Table 1. Basic characteristics.** Basic characteristics of the 40 included cadavers (median, interquartile range IQR).

**Table 2. Cases with a pseudolesion.** Cases with a pseudolesion on the non-contrast-enhanced postmortem CT on the right side of the falciform ligament.
