# Supplementary material for: Pseudolesion in the right parafissural liver parenchyma on CT: The base is found in embryology and collagen content
Source: PLoS One. 2020 Jan 27;15(1):e0221544. doi: 10.1371/journal.pone.0221544 (PMC6984698; doi:10.1371/journal.pone.0221544)
Supplement: Attachment — Submitted filename: response to reviewers.docx [file pone.0221544.s003.docx]

**Response to Reviewers**

Rebuttal Letter

Leonidas G Koniaris, MD
Academic Editor

PONE-D-19-22279
Pseudolesion in the right parafissural liver parenchyma on CT: the base is found in embryology and collagen content.
PLOS ONE

Dear editor,

Thank you for your thorough review of our manuscript and thank you for welcoming a revised version.

You have advised us to edit the discussion for readability and clarity, without requirement for additional data. The reviewer advised us to make the discussion more succinct. We agree that this would help to improve the manuscript and we have applied this. In the track& trace version you can see the adjustments made in the discussion, that make the discussion more readable and succinct.

Further you have advised financial updates (that we have none) and journal style requirements (that we have all applied according to the Plos ONE style). Also I have enclosed a FAIR dataset that is fully anonymized that can be published with the manuscript. Numbers have been

Journal requirements 1: the title, authors and main body have been adjusted.

Journal requirements 2 and 3: A FAIR dataset is now added. We have supplied a minimal dataset with rounded numbers (age) to diminish any recognizability in the context of privacy.

Reviewer:

Suggestion 1.

The reviewer argues that the embryological images do not add much to the discussion. We disagree. It is one of the main messages of this manuscript that the cause of a supposedly abnormal anatomy can be found in embryology. We find it important to show the embryological evaluation of the umbilical veins to support our hypothesis. Therefore we wish to leave the images as these are, illustrating the paragraph Etiophysiology in the Results.

Suggestion 2.

The reviewer gives a suggestion how to further study the liver focal fatty sparing, namely by biopsy during laparotomy. We have added this to the discussion and thank the reviewer for his valuable addition.

We have adjusted the discussion to make it more succinct. We thank the reviewer for his thorough review of our manuscript and for helping us to improve it.

We look forward to your decision concerning our manuscript. Thank you for your important contribution to this study, concerning the liver from embryology to postmortem, in the matter of the parafissural pseudolesion. Our results will be helpful to the everyday work of radiologists and hepatologists all over the world.

Sincerely,

Willemijn M. Klein

On behalf of the authors.
